# Lived experience perspectives guiding improvements to the Systematic Tailored Assessment for Responding to Suicidality protocol

**DOI:** 10.3389/fpsyt.2023.1074805

**Published:** 2023-07-07

**Authors:** Jacinta Hawgood, Carmen Betterridge, Kairi Kõlves, Bronwen Edwards, Susan H. Spence, Ella Arensman, Diego De Leo, Tamara Ownsworth

**Affiliations:** ^1^Australian Institute for Suicide Research and Prevention, World Health Organization Collaborating Centre for Research and Training in Suicide Prevention, School of Applied Psychology, Griffith University, Brisbane, QLD, Australia; ^2^School of Applied Psychology and Menzies Health Institute Queensland, Griffith University, Brisbane, QLD, Australia; ^3^Suicide Risk Assessment Australia, Sydney, NSW, Australia; ^4^Roses in the Ocean, Brisbane, QLD, Australia; ^5^School of Public Health, College of Medicine and Health, University College Cork, Cork, Ireland; ^6^National Suicide Research Foundation, Cork, Ireland

**Keywords:** suicide prevention, qualitative research, lived experience of suicide, co-design of assessment protocols, psychosocial needs-based assessment, risk assessment

## Abstract

**Background and aims:**

Suicide risk assessment protocols have traditionally been developed by clinical or research experts in suicidology, with little formal involvement of those with a lived experience of suicide. This study broadly aimed to seek lived experience perspectives of the Systematic Tailored Assessment for Responding to Suicidality (STARS) protocol A further aim was to elicit lived experience suggestions for wording and language used in the existing items within sections of the STARS protocol (STARS-p).

**Method:**

Participants were 33 adults (Female = 64%) with a lived experience of suicide, who attended a virtual research workshop at the National Lived Experience of Suicide Summit (2021). After being educated about STARS-p, participants provided their overall perceptions of STARS-p as well as suggestions for rewording and language use across the sections of STARS-p. Their responses were gathered using a virtual online platform for live electronic data collection. A three-phase process of qualitative content analysis was used, engaging both inductive and deductive approaches to explore study aims one and two, respectively. The Consolidated Criteria for Reporting Qualitative Research was followed to enhance quality of reporting.

**Results:**

Qualitative content analysis of participants’ views of the STARS-p reflected three main categories, namely, STARS philosophy; What STARS aspires to; and Continuity of care and meeting needs. Responses characterized participants’ perceptions of the core purpose of STARS-p and ways for refining or adapting it to suit diverse needs and settings. Based on deductive content analysis, suggested modifications to wording of items and additional items to extend sections were identified.

**Conclusion:**

The study yielded novel perspectives from those with a lived experience of suicide, which will inform improvements to the next edition of STARS-p. The STARS training (required for licensed use of the protocol) will be updated accordingly, in line with these results.

## 1. Introduction

Suicide prevention approaches are increasingly recognizing the importance and value of including lived experience of suicide in the design, development, delivery, and evaluation of initiatives (1–4). Gaining such perspectives is critical because such individuals have direct experience of the issues being targeted by prevention, and may therefore have unique insights regarding optimal approaches to assessment and support. Lived experience has been defined as “having experienced suicidal thoughts, survived a suicide attempt, cared for someone through suicidal crisis, or been bereaved by suicide” (5). Engaging people with lived experience in suicide prevention interventions and program development is now considered paramount for advancing suicide prevention efforts in Australia (6). The roles of lived experience experts may vary from involvement in “project co-design, development, implementation, and evaluation of programs, policy advising, awareness-raising speaker engagements, undertaking collaborative research, and supporting others who are suicidal or bereaved by suicide through peer work” (7). However, scant attention has been given to the role of lived experience in design or refinement of suicide risk assessment approaches specifically.

Comprehensive assessment and monitoring of suicidality are emphasized as key elements of suicide prevention worldwide (8). While a plethora of suicide risk assessment tools have been developed for either screening of suicidality or comprehensive suicide risk assessment (9), there are very few systematic, semi-structured interview-based protocols worldwide (10–13). In Australia, the STARS-p (14) (see Supplementary Material 1 for protocol description) is the only semi-structured, psycho-social interview, developed with the key objective of supporting mental health professionals (MHPs) to collaboratively explore and understand individuals’ subjective account of their experience of suicidality and develop a commensurate safety plan response (15). The STARS-p is a semi-structured interview that does not attempt to predict suicide but takes a structured professional judgment (SPJ) approach (16) through empirically informed questions regarding suicidal state (Part A), psycho-social risk (Part B) and protective factors (Part C) to inform client care plans. However, feedback on the utility and application of STARS-p to date has been received only from MHPs, the main users of the protocol in practice (17).

Overall, clinicians highlighted both advantages and barriers to using STARS-p in practice. Key advantages related to the practical and flexible administration of the protocol for tailoring to different client presentations. The main barriers included the time-consuming nature of administration and potential lack of suitability of structured interview approaches for some populations (e.g., First Nations peoples). Practitioners’ main recommendations for improving the utility of the STARS-p included expansion of space in the protocol for notetaking, a digital based version for easier record keeping and updating of client data, and ongoing training for STARS-p administration in practice.

While practitioner perspectives are valuable for informing refinements to the STARS-p to support greater uptake in practice, suicide risk assessment (SRA) approaches also need to be acceptable to those with lived experience of suicide (17). SRA protocols have traditionally been developed by clinical and or research experts in the domain of suicidology. Until recently, there had been no published literature on the development nor review and revision of SRA protocols by those with a lived experience of suicide. Only one study by Dimeff et al. (18) has involved those with a lived experience of suicide to inform assessment processes, with such perspectives sought on the adaption of an existing assessment and management protocol to online-digital form (18). Notably, authors in the suicide prevention literature have been increasingly advocating for engagement and contributions of people with lived experience, with co-design of suicide-related prevention and treatment initiatives considered optimal (19–22). The value of lived experience in design and development of assessment protocols specifically cannot be understated; such expertise in item construction and use of appropriate and safe language is critical for creating engaging and empathic communication processes between interviewer and client. Some existing assessment tools have been criticized for their spurious item construction as well as lack of meaning for those whose needs they are designed to meet (23). While engaging those with a lived experience of suicide in assessment protocol design is important, some challenges have been identified in the research process for undertaking this important work. These include the duration of the co-production and other co-design components of protocol or intervention development, fears and pre-conceptions around tokenism and ensuring appropriate reimbursement, and potential for activation or rumination of suicidal thoughts in participants (24). Nevertheless, involvement of those with lived experience of suicide may improve the construction of appropriate SRA enquiry probes and administration processes (23). Specifically, lived experience experts’ views on appropriate language use, terminology, and formatting of SRA questions may enhance meaningful exploration of this deeply complex internal experience. For example, the ‘desire’ and ‘capacity’ for suicide must be asked in a way that differentiates between them (23). Often people will experience ongoing suicidal thoughts without any intent or capacity to enact a plan to take their life, and in fact, may associate feelings of calm to these thoughts which may paradoxically protect them against taking their life (25). Without input and guidance from those with lived experience, MHPs may miss important situational and contextual cues, and personal narratives that help to inform both the assessment of a person’s suicidal state as well as their psycho-social needs and safety.

An important element of STARS-p is its client-centeredness in which the contributing and precipitating factors to suicidality are identified from the perspective of the client, to inform the formulation of a management plan. In order to enhance the capacity of STARS-p to achieve this goal, the current study invited the contributions of those with a lived experience of suicide into the modification and refinement process of the next edition of the STARS-p. The overall goal was to formally involve and gather the collective contributions and perceptions of those with a lived experience of suicide to inform the improved item construction, terminology, administration processes and refinement of the STARS-p (14).

Accordingly, the current study’s aims were to:

1. Explore the perceptions of those with a lived experience of suicide regarding the STARS-p.
2. Elicit perceptions of, and suggestions for, refinement and improvement of existing wording and language associated with STARS-p, Parts A–C.

## 2. Methods

### 2.1. Participants, procedure and design

Participants (*N* = 33) who had a lived experience of suicide, in terms of their own suicidality, surviving a suicide attempt, losing a loved one to suicide, or caring for someone in suicidal distress (5), were invited to volunteer their insights and perceptions as part of an interactive, 3.5-h STARS-p workshop, hosted by Roses in the Ocean as part of the National Lived Experience Summit, 2021 (Australia). The workshop aimed to gather perceptions from those with a lived experience of suicide about the STARS-p. While it is clear that the perceptions of those with living or lived experience of suicidal thoughts or behaviors were critical to addressing the key aims of this study, gaining the perceptions of those who are bereaved by suicide and/or those who have cared for those in suicidal distress was also considered valuable. These individuals may have useful insights into the experiences of suicidality of their loved ones as they are often in closest contact with and supportive of them during their suicidal distress, as well as most frequently able to observe characteristics and experiences prior to suicide and/or suicide attempt (26). Furthermore, in many situations (we recognize, not in all situations), they have been an integral part of their loved one’s safety planning or support mechanisms, which are key components of the STARS-p management plan.

Roses in the Ocean advertised the workshop which was one of several workshops delivered as part of the larger Summit via multi-media platforms and other stakeholder networks within the Australian suicide prevention sector, encouraging all stakeholders in suicide prevention to attend; but specifically, those with a lived experience of suicide.

The types of lived experience of the sample reported were not mutually exclusive and included: experienced suicidal thoughts (79%), survived a suicide attempt (73%), cared for a loved one through a suicidal crisis (64%) and bereaved by suicide (39%). Table 1 presents the proportions of multiple types of lived experiences of participants, followed by the overall proportion for each type of lived experiences of participants. The predominant reasons for participation included: ‘learning/enhancing knowledge and skills’ (42%), ‘to contribute lived experience for better assessment/protocol development’ (24%) and ‘curiosity/interest in the STARS-p’ (15%).

**Table 1:** Proportions of lived experience for participants (*N* = 33).

Type of lived experiences	*n*	Proportion
Experienced suicidal thoughts + survived a suicide attempt	8	24%
Experienced suicidal thoughts + survived a suicide attempt + caring experience	8	24%
Experienced suicidal thoughts + survived a suicide attempt + caring experience + bereaved by suicide	7	21%
Caring experience	3	9%
Experienced suicidal thoughts + caring experience + bereaved by suicide	2	6%
Bereaved by suicide	2	6%
Experienced suicidal thoughts + Bereaved by suicide	1	3%
Caring experience + bereaved by suicide	1	3%
Survived a suicide attempt	1	3%
Total type of lived experience		
Experienced suicidal thoughts	26	79%
Survived a suicide attempt	24	73%
Cared for a loved one through suicidal crisis	21	64%
Bereaved by suicide	13	39%

A participant information pack was provided at workshop registration several weeks prior to the Summit. The information pack included study rationale, aims, design and expected outcomes of the workshop, participation tasks, confidentiality and informed consent issues, including the right to withdraw voluntarily, and safety and support provisions. Also included was information indicating that the lead investigator of the study is the lead author of the STARS-p. Roses in the Ocean ensured peer-support, debriefing and professional support systems were available from commencement of the workshop to after the Summit event. Regular safety and support ‘check-ins’ were undertaken throughout the workshop by facilitators.

The consent form was an attachment within the workshop information pack and included space for participant demographic details to be recorded and provided back to Roses in the Ocean as part of the workshop registration process. A de-identified spreadsheet listing consenting participants and associated demographic details was provided by Roses in the Ocean to the research team. Upon receipt of the signed consent form, each participant was emailed a schedule of the questions that would be asked of them in the workshop. During the workshop participants were asked to type directly into the virtual online whiteboard and Slido platform aligned with the webinar portal.

A qualitative descriptive design was used to answer the research questions. All procedures were approved by the Griffith University Human Research Ethics Committee (Ref number: 2021/651/HREC). The Consolidated Criteria for Reporting Qualitative Research (27) guided the methods and reporting (see Supplementary Material 2).

### 2.2. Data collection

The workshop was designed to gather participant responses on: (a) overall perceptions of STARS-p and (b) perceptions about the wording or suggestions to improve the existing interview domains and associated items of the protocol – STARS Parts A–C (including the language used for interviewer probes within these Parts). Due to COVID-19 in-person contact restrictions the workshop was delivered online, using a real-time interactive process via a virtual platform. For the full details see Supplementary Material 3.

The workshop was delivered by two lead facilitators, authors JH (female, registered clinical psychologist) and CB (female, registered psychologist) who have 25 years and 21 years of registration as psychologists, respectively. The lead facilitators did not know the workshop participants personally. Support facilitation was provided by author BE (lived experience expert, and CEO of Roses in the Ocean), with additional support provided by another Roses in the Ocean member. The facilitators first presented STARS-p; its philosophy, purpose, general administration process, and the specific questions within each STARS-p section (Parts A–C). Facilitators then invited participant brainstorming around research questions, firstly in one of four breakout rooms, each with approximately 8 participants, and then in the subsequent larger group workshop room. No participants left the workshop; thus, it is assumed all participants engaged in the workshop through brainstorming and/or scribing their responses to questions.

Research questions were administered using the Vimeo platform (powered by AVPartners) virtual whiteboard (for small group responses) and Slido (for larger group responses). All participants were given the opportunity to answer each question, firstly via virtual whiteboard in smaller breakout rooms and subsequently, using Slido in the large group forum during the discussions in the larger group. The specific questions (labeled Q1–Q4) of the workshop appear in Table 2.

**Table 2:** STARS-p workshop, research questions.

Questions	
Q1	What are your overall views on the STARS Protocol?
Q2	What items, if any, do you think could be worded better in PART A of the STARS protocol?
	If relevant, what is an alternative way to ask this question/s? *(Consider appropriate language use, while also obtaining direct information on the domain of enquiry)*
Q3	What items, if any, do you think could be worded better in PART B of the STARS protocol?
	If relevant, what is an alternative way to ask this question/s? *(Consider appropriate language use, while also obtaining direct information on the domain of enquiry)*
Q4	What items, if any, do you think could be worded better in PART C of the STARS protocol?
	If relevant, what is an alternative way to ask this question/s? *(Consider appropriate language use, while also obtaining direct information on the domain of enquiry)*
	Do you have any further comments about the STARS Protocol?

### 2.3. Data analysis

**Figure 1:**
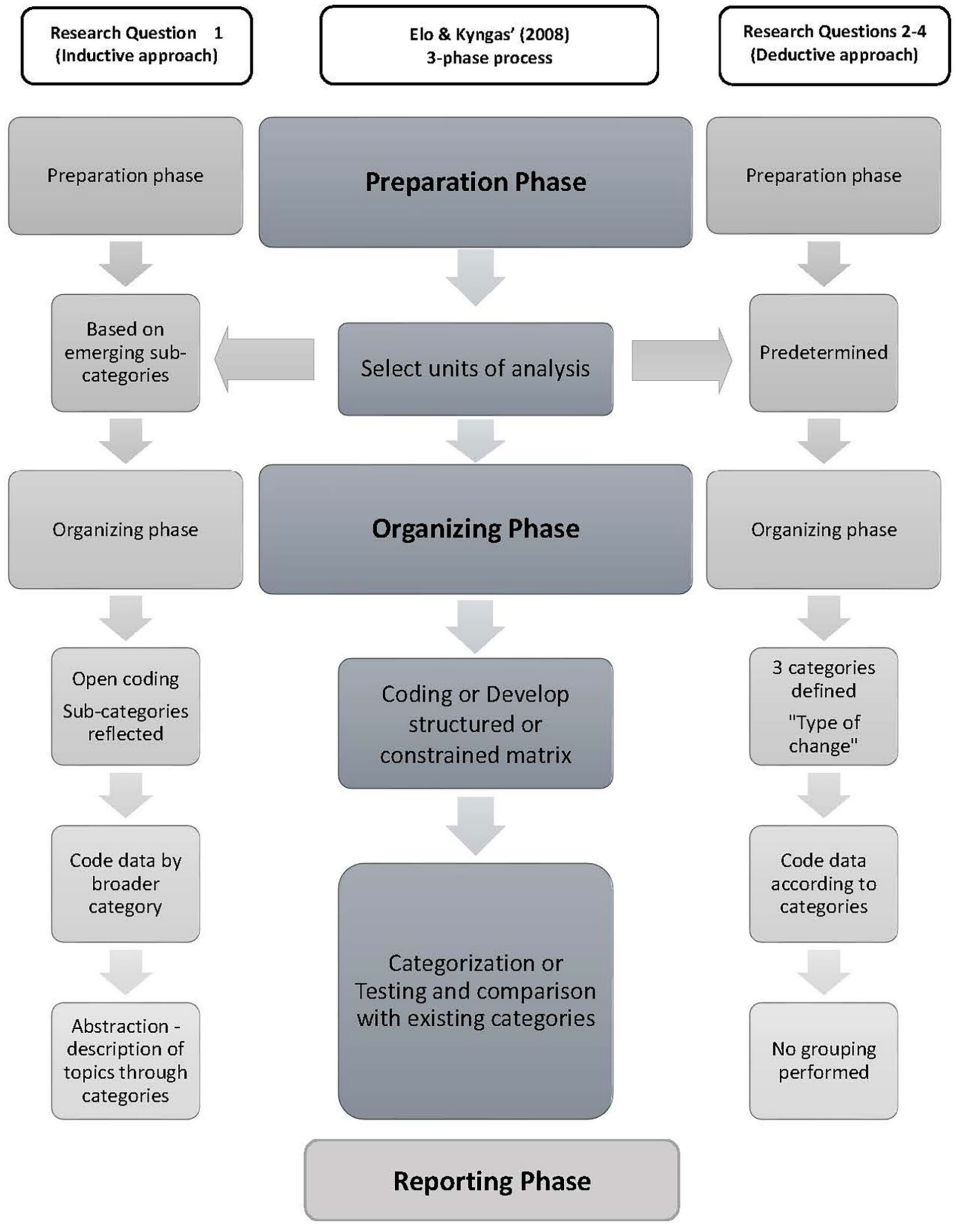
Elo and Kyngas’ (29) qualitative content analysis framework: Inductive and deductive approaches using three phases of analysis.

Prior to analysis, participant responses from the two mediums of data collection (virtual whiteboard and Slido responses), were merged into one Microsoft Excel data file and were screened to ensure participant responses were correctly placed under the response headings for Questions 1–4. Any text gathered from participant responses (such as story-telling and self-disclosures of experience) that were deemed to be unrelated to the question at hand were removed from analysis but noted (28). To explore lived experience perspectives of the STARS-p we employed Elo and Kyngas’ (29) three-phase process of qualitative content data analysis, as follows: (1) Preparation of data, which included data screening to determine the best unit of analysis based on responses, (2) Data organization, which included grouping the data units into sub-categories and categories, and finding suitable labels for them and (3) Resulting, which is reporting the analyzing process. Figure 1 presents the three-phase approach used for qualitative content analysis following Elo and Kyngas’ (29) model, including both inductive and deductive approaches.

#### 2.3.1. Phase 1

According to Elo and Kyngas (29), phase 1 (preparation phase) is similar for both inductive and deductive approaches. For the analysis of Q1 (inductive approach), data were independently reviewed by author TO and a senior research assistant; neither of whom is an author of the STARS-p. This review process involved determining the best unit of analysis based on the level of detail in responses (e.g., words, themes, sentences and the like), as well as reading and re-reading the raw data responses and determining the grouping of similar words or groups of words/phrases together as the unit of analysis. Preparation for analysis of Q2-4 (deductive approach), involved the decision to develop a coding categorization matrix based on existing interview categories within the STARS-p, so that the researchers could collate participant perceptions of existing interview items in the protocol. Therefore, Phase 1 included determination and preparation of unit of analysis (Q1), and the use of structured categories for coding of responses in Q2–4.

#### 2.3.2. Phase 2

We used the inductive approach for addressing aim one of this study (Q1, exploring overall perceptions about STARS-p), while a deductive approach was deemed most suitable for addressing aim two of the study (Q2-4, eliciting perceptions about suggestions for wording and language improvements to existing interview items within the protocol). Regarding this latter aim, the workshop facilitators educated the participants about STARS-p, including the protocol domains of enquiry and the existing interview questions within the STARS-p (Parts A, B and C).

For the analysis of Q1, organizing the qualitative data involved open coding in an excel data file (by typing comments while reading through the data) (conducted by TO, senior research assistant and JH), grouping of data into sub-categories, and then grouping the data into broader-level categories (29) (conducted by JH and TO), followed by abstraction or the application of a general label or description relevant to the content of the category. The authors (JH and TO) collaboratively classified sub-categories into groups in terms of how they ‘belong’ together, rather than just being similar or related to each other (29). Therefore, there were no pre-existing categories or coding matrices applied to the data, allowing the overall views of the STARS-p to emerge, with sub-categories being created based on the participant’s responses. Two authors (JH and KK) randomly reviewed the responses associated with different sub-categories, and any disparities that arose in coding were discussed and amended following agreement (see further detail on managing disagreements below). Authors BE and CB reviewed and contributed to interpretation of coding of responses into sub-categories and categories. The final step in phase 2 involved the abstraction process, where final labels (including meaningful content-descriptions of the categories and the main category) were formulated, involving all authors.

The organizing phase for the analysis of Q2-4, involved development of the structured categorization matrix consisting of a list of the existing STARS-p domains of enquiry, and the associated questions for Parts A, B, and C in the order that they appear in the protocol. Adjacent columns were created for inclusion of the participant comments/responses, under alternative suggested wording categories reflecting the suggested ‘type’ of change: (1) addition, (2) reorder and (3) reword. The coding matrix was therefore constrained (or structured) in that no new sub-categories were created from the responses. Rather, each response was coded as suggesting an *addition*, *reorder* or *rewording* of the existing category and associated items in STARS-p. Using this categorization matrix, data were then reviewed by JH, TO, KK, BE, CB and a senior research assistant for correspondence with the developed categories. The authors discussed the importance of analyzing manifest content only, as opposed to latent content of responses, since the main aim was to interpret that which was literally presented as language and terminology suggestions for refinement or modification of existing STARS-p items. The frequency of responses from each domain of enquiry, and the associated category suggestions were calculated to support analysis and interpretation.

Given that two of the team members are also creators of STARS-p, to ensure rigor of the analysis (30, 31) research team members with lived experience (BE) and no direct involvement with STARS-p (senior research assistant) were involved in the analysis (Phases 1 and 2 for inductive and deductive approaches: categorization of the responses, label-selection, verification of the coding matrixes). Any bias was minimized by involving multiple coders during all levels of analysis, and initial data review and grouping into codes was done by team members who were not involved in the workshop (TO and KK). No participants were invited to provide feedback on the findings.

Only the inductive approach (used for analysis of Q1) required planned strategies for managing any disagreements between coders given that our deductive approach for analyzing Q2-4 was directly guided by the categorization matrix which was structured according to existing content domains within the STARS-p. We used open discussion and negotiation between coders for management of any disagreements. In the shared excel data file of participant responses to Q1, a column was created for each coder to note discrepancies. Discrepancies occurred for 14 (89%) of all codes. Subsequent discussion between coders was used to gain understanding of their respective interpretations, to understand the rationale and meaning behind allocation of responses to codes until consensus was reached. There were no instances where consensus was not achieved.

### 2.4. Rigor and trustworthiness

To enhance the rigor of the analysis, we undertook a reflexive process and involved multiple researchers in the data analysis processes. The research team included a combination of expert researchers in suicidology as well as lived experience experts, increasing the diversity of examination of alternative explanations. We also used continuous checking and review processes, allowing for ongoing opportunities to determine representativeness of the data and fit between the sub-categories and categories.

## 3. Results

### 3.1. Participant characteristics

A total of 33 participants participated in the virtual workshop and provided responses. Participants were mostly female (64%), aged between 30 and 59 years (78%), and of Non-Indigenous Australian and Caucasian ethnicity (64%).

### 3.2. Question 1 – what are your overall views of the STARS protocol?

**Figure 2:**
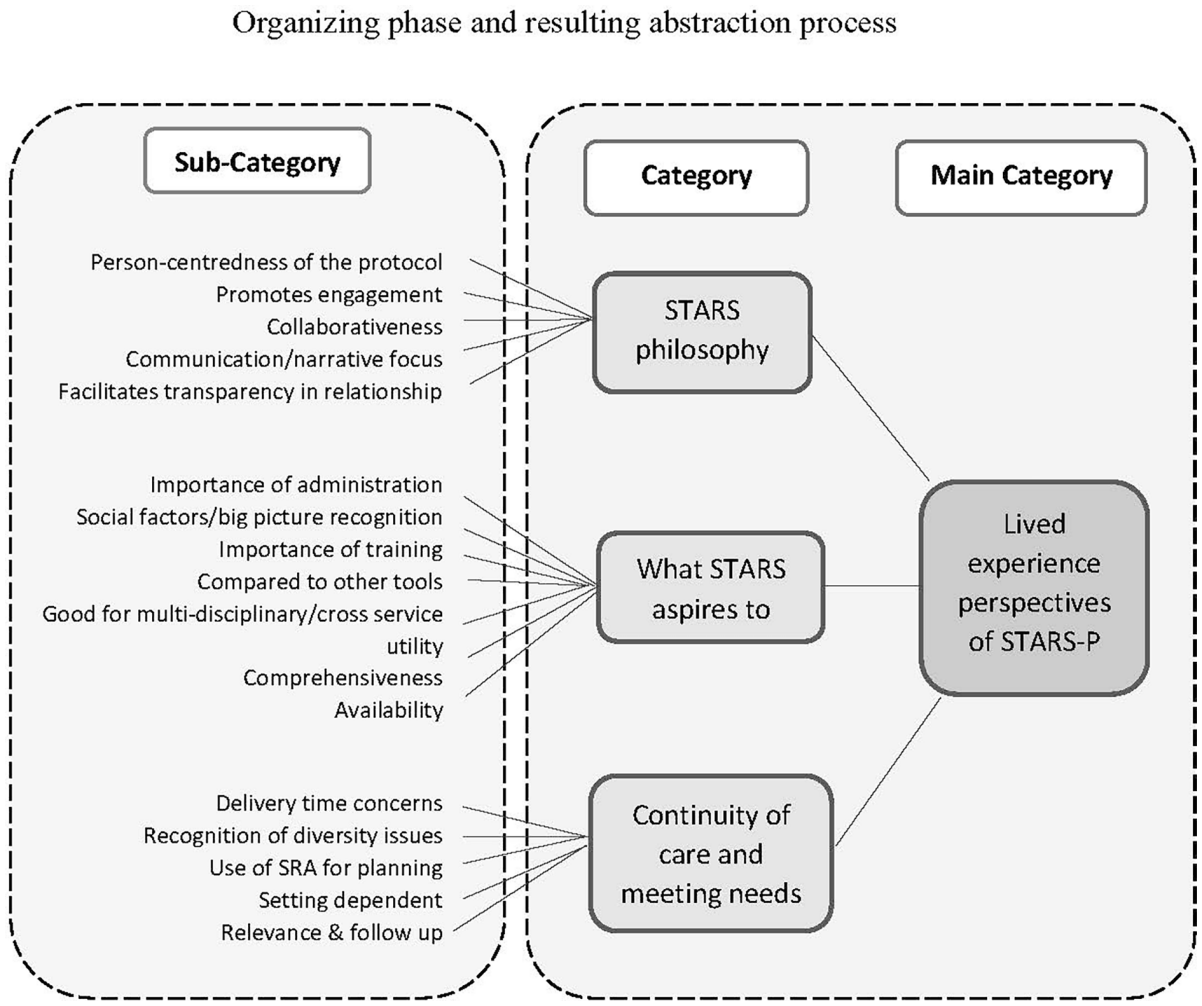
Inductive analysis approach to Q1 using Elo and Kyngas’ (29) framework of analysis.

Figure 2 shows the sub-categories arising from the data preparation phase as well as the associated three categories of meaning resulting from the final abstraction process: 1. STARS philosophy; 2. What STARS aspires to; and 3. Continuity of care and meeting needs. Table 3 presents exemplar quotes associated with the sub-categories and categories. These collectively underpin the main category of lived experience perspectives of STARS-p.

**Table 3:** Q1 results: lived experience perspectives of STARS-p.

Category	Sub-categories	Quotes
STARS philosophy		
	Person-centredness of the protocol	“Allows deeper conversation with person and covers comprehensive list of topics, I like that it’s person-centred and allows someone to tell their story and assess how personally significant different items are….”
	Promotes (or challenges) engagement	“As a risk assessment tool goes, it seems OK. The focus of any risk assessment should be measured by how the person receiving the care feels about that care. Its efficacy would depend largely on the person delivering it. It needs to have a co-form around practicalities of connection to be able to deliver this and capture the information effectively.”
	Collaborativeness	“Truly a mutual collaborative protocol which honors the expertise of the lived and living experience of the help seeker. Co-authoring of information, analysis and strategies for protecting the individual empower the resilience and other strengths of them and their naturally determined supports….”
	Communication/narrative focus	“…Self-awareness, analysis and construction of safety planning is inherent in choice and control over the life that is reflected in the narrative of the individual - the worker is witness, reflection guide and coach. How powerful this might be if the worker has Lived Experience to deepen the connection and sense of belonging.”
	Facilitates transparency in relationship	“Liked how it’s open transparent / note taking – and about them seeing we GET THEIR STORY, not just some table/grid/rating.”
What STARS aspires to	Importance of how protocol is administered (how questions are asked, introduction and consent process, and giving power to the person).	“Its efficacy would depend largely on the person delivering it. It needs to have a co-form around practicalities of connection to be able to deliver this and capture the information effectively.”
		“It does not go far enough AWAY from risk assessment. I feel the person needs to be in control of their story. The interviewer is the one with the power - give that power to the person.”
		“Like most things, I think this could be a great tool or a poor tool depending on how it is used. I can see that the goal is for it to not be a checklist, but it would still very much depend on the way it’s administered as to whether it ends up feeling like a check-list or not.”
	Social factors/big picture recognition	“I love the inclusion of socioeconomic risk factors. However, how do you address these? e.g., Someone is there due to financial issues- are you going to find them help to address these issues?”
		“Recognizes systemic issues / social justice aspects around mental health. Removes sense of “blame” on the person. Reveals mental health as often a societal issue over and above the individual. I think the protocol is great.”
	Importance of training	“Proper training for the staff is imperative - can see that the process could also de-escalate the crisis exponentially - Good for everyone concerned - personalized it for the consumer….”
	Comparison to other tools	“Very comprehensive and very person centred; better than existing tools and very important for all workers - clinical and non clinical”
		“Amazing. Clear, poignant and relevant questions. Existing protocols are patronizing, not person-centred and largely useless because they do not reveal enough about what is actually going on for the person nor do they create a sense of confidence in the process and therefore people often lie or obscure their responses….”
	Multi-disciplinary/cross-service utility	“I think that the best potential for success with this tool is to look at the roles of the people that are going to utilize it with individuals. There are roles like mine that are community-based suicide prevention - have the time to have the conversation and follow up in the short and longer term. And importantly it’s the connection and ability to work collaboratively (with consent of course) to keep GP’s etc. involved”
	Comprehensiveness of protocol	“I think the explicit nature of the questions is important. I like the flow of the protocol as well. Great that it is asking about everything in a person’s life.”
	Availability of protocol	“I agree but I’m concerned about how readily available this protocol is. For example, how many people know about it?”
Continuity of care and meeting needs		
	Delivery time concerns	“…Completing the document to the quality needed relies on having enough time and space for the person to feel comfortable & willing to share which could be a real challenge in busy services.”
	Recognition of diversity issues	“…there is also a lack of LGBTIQAP+ recognition in it. Sexual identity and gender identity are two different things and come with different risks and safety factors.”
		“…It does not really address intersectionality that exists between disadvantaged groups (LGBT+, disability, multicultural, etc.) and how they impact suicidality, also somewhat inaccessible for ND people in some questions (a common problem for psych measures in general)….”
		“…For LGBTIQ populations it needs to be introduced properly to actually have people open up and confide in the person interviewing. I.e., often useful to state pronouns on intro to person which allows a safe space for the person to confide if they have a diverse sexuality or gender”
	Use of SRA information for planning	“Ideally it could be used as a collaborative tool and shared between services so there’s an actual plan to address the issues raised - it could be hard for the person to identify all these needs and not have anyone help them actually meet them.”
	Setting dependent	“I cannot see this ever happening in an ED, but I think it would be great in a Safehaven type setting.”
	Relevance and follow-on to other services	“It’s a great risk assessment tool, but I’m still concerned about support & follow-up as I feel this would still be at the clinician’s/health facility’s/service’s discretion”

In relation to the category STARS philosophy, participants broadly perceived that the protocol questions are relevant to the person who is seen as central and expert and that it promotes engagement, collaboration, and transparency. Participants emphasized the importance of person-centredness with the protocol facilitating in-depth conversation for the person to tell their story. For example, “Allows deeper conversation with the person and covers a comprehensive list of topics” and, “I like that it’s person-centred and allows someone to tell their story and assess how personally significant different items are.”

The second category depicts participants’ perceptions of aspirational elements in terms of how the protocol is administered and used, and how it can be more effectively used alongside training and across multiple disciplines. For example, “Like most things, I think this could be a great tool or a poor tool depending on how it is used. I can see that the goal is for it to not be a checklist, but it would still very much depend on the way it’s administered as to whether it ends up feeling like a checklist or not”, with, “Recognizes systemic issues / social justice aspects around mental health. Removes sense of “blame” on the person. Reveals mental health as often a societal issue over and above the individual. I think the protocol is great.”

The third category reflects participants’ views on the importance of continuity of care and adaptations to the protocol to meet the different needs of diverse populations and its suitability of applications in different settings. Participants emphasized how optimal use of the protocol to meet client needs requires time, recognition of diversity and collaborative service planning. For example, “Ideally it could be used as a collaborative tool and shared between services so there’s an actual plan to address the issues raised….,” and “there is also a lack of LGBTIQAP+ recognition in it. Sexual identity and gender identity are two different things and come with different risks and safety factors.”

### 3.3. Questions 2–4 – on alternative wording to parts A–C

Tables 4–6 depict the summary of findings for participant suggestions to changes for Parts A, B, and C in line with the categorization matrix of STARS-p domains of enquiry, in order of item presentation. Also conveyed in these tables is the type of change, summarized suggestions for the change, and related quotes. As seen in Table 4, there were suggestions for changes in eight domains of enquiry within Part A of STARS-p listed in the matrix, with ‘death ideation’ receiving the most suggestions (*n* = 11). The most prominent type of suggestion across all domains in Part A related to rewording of items. No responses were received for the STARS-p domain ‘current mental state’.

**Table 4:** STARS-p PART A – summary of suggestions for improvement of item wording, language, and ordering.

Domain of Enquiry	Type of change			Summary of suggestions	Example suggestion (quote)
Death ideation	Addition	Reorder	Reword	Alternative wording suggestions focused on more clearly delineating thoughts of death from thoughts of suicide.	“Have you thought about your death or dying in general, such as by natural causes, but not suicide specifically?” Reword for: Have you wished to be dead (i.e., by natural causes?)]
	0	0	11		
Suicidal thoughts	Addition	Reorder	Reword	Question on ‘intensity’ and temporal nature of ideation. Reduce jargon and ask less directly about suicide.	“have the thoughts been increasing recently?” (Addition)
	1	0	1		“Might read better the other way around (to reduce jargon, but still then mention “suicide” at the end of Q) [ask about ending your life first]” [Reword for: Have you thought about suicide/ending your life (recent, past, current)?]
Plans/intent	Addition	Reorder	Reword	Alternative wording suggestions for ‘planning’, focused on need for clarity, and lay person language, while ‘communication to others’ focused on relational context of plan (e.g., alone or around others).	“2 questions sound much the same – a bit confusing how things are ordered.” (Reorder)
	0	1	5		“‘Have you ever made a plan to end your life? Not just ‘have you ever made a plan?” (Reword from: Have you ever made a plan in the past?)
Access to method	Addition	Reorder	Reword	Alternative wording focused on more specific questions around method use.	“How did you come to this method? To find out if they have actually researched the lethality” (Addition)
	2	0	2		“Which methods do you have access to? (not just one specific method).” (Reword from: Do you have access to any specific method?)
Previous attempt	Addition	Reorder	Reword	Alternative wording suggestions for prior method-use, type of help sought, and acting impulsively focused on need for sensitivity around client perceived judgments.	“When you last felt like this how did you get access to help/support?” (Addition)
	1	0	4		“Could be I wonder if you think that you acted suddenly/without thought?” (Reword from: Do you think you acted impulsively?)
Previous non suicidal self injury (NSSI)	Addition	Reorder	Reword	Alternative wording suggestions for acts of NSSI, concerned seeking more information or more sensitive questioning around self-harm.	“Add why? – to, have you engaged in any acts of non-suicidal self-injury before?” (Addition)
	1	0	5		“Have you hurt or harmed yourself before?” (Reword from: Have you engaged in any acts of non-suicidal self-injury before?)
Psychiatric care/help	Addition	Reorder	Reword	Alternative wording suggestions focused on eliciting experience of care from services.	“Ask if there has been trauma related to the medical system that may prevent/discourage access of care as a barrier” (Addition)
	1	0	3		“Do you have / Have you had / Are you satisfied with your psychiatric care?” [Reword from: Have you received mental health treatment in the past? (Specify experience –negative/positive/perceived stigma, etc.)]
Current mental state	0	0	0		
All/admin	Addition	Reorder	Reword	Suggestion to move sensitive items related to death later in Part A.	“Also feel like the first part of line of question might be better in the middle. Not at the start.” (Reorder)
	0	1	0		

Table 5 presents the findings of Part B, for which 13 of 15 STARS-p domains of enquiry received suggestions. The domain ‘sexual identity/orientation’ received the highest number of participant total responses (*n* = 19) for suggested changes, followed by ‘sexual/physical/emotional abuse’ (*n* = 11) and ‘mental health condition’ (*n* = 10). All domains included ‘rewording’ suggestions, but participants also requested ‘additions’ within the domains ‘sexual identity/orientation’ (*n* = 9), ‘drugs and alcohol’ (*n* = 3) and ‘health’ (*n* = 1). Three further responses for ‘additions’ to items/questions were not indicated for any specific domain of enquiry but were nevertheless related to Part B feedback.

**Table 5:** STARS-p PART B – summary of suggestions for improvement of item wording, language, and ordering.

Domain of enquiry	Type of change			Summary of suggestions	Example suggestion (quote)
Home	Addition	Reorder	Reword	Alternative wording suggestions concerning ‘safety’ and temporal experience.	“Are there times you feel unsafe at home?”
	0	0	2		
Education/employment	Addition	Reorder	Reword	Suggestions for reordering of items on: work, occupation, school. Rewording suggestions focused on positively reframing questions on work roles.	“Are you working at the moment? What is your occupation? How are things at school/work?” (Reorder)
	0	2	3		“Are you working (paid or unpaid) at the moment?” (Reword from: Are you not working at the moment?)
Sexual/physical/emotional abuse	Addition	Reorder	Reword	Suggestions for additional items for trauma (not abuse) and generational experiences. Rewording suggestions focused on seeking clients’ view of these items and their impacts/implications.	“[is there] any generational trauma etc.?” (Addition)
	3	0	8		“Have you experienced trauma, abuse or neglect in the past or currently” [Reword from: Have you been abused (past) or are you currently being abused?]
Marital/ *De Facto* relationship	Addition	Reorder	Reword	Rewording suggestions focused on questioning ‘any relationship’ issues from the outset.	“Are you experiencing any relationship issues (not limiting to breakup) “(Reword from: Are you experiencing a relationship breakup/separation?)
	0	0	4		
Financial problems	0	0	0		
Mental health condition	Addition	Reorder	Reword	Suggestions for ‘additions’ concerned obtaining clients’ view and impacts of their diagnosis. Rewording suggestions focused firstly on more broadly asking about mental health or ill-health.	“Do you agree with your diagnosis? What are your thoughts on your diagnosis?” (Addition)
	2	0	8		“Have you been formally diagnosed with a mental health condition ^ or thought that you could be?” (Reword from: Do you have a psychological problem?)
Health	Addition	Reorder	Reword	Suggested additional content concerned expanding questions in this domain and rewording focused on more direct enquiry.	“[Part B] may need to be expanded” (Addition)
	1	0	2		“Have you been diagnosed with a health condition?” (Reword from: How is your physical health?)
Sexual identity/orientation	Addition	Reorder	Reword	Suggested additions for gender identification item. Rewording suggestions concerned sensitivity, while reordering focused on questioning impacts of negative experiences first.	“How would you describe your gender identity?”; “What pronouns do you use?” (Addition)
	9	1	9		“If you know it’s not the sexuality/home life/money etc. but its effect on the person, why do not you flip those two elements around and focus on the pain, loss, stress, marginalization etc.?” (Reorder)
					“What has your experience of your sexuality and gender been like?” (Reword from: Are you comfortable with your sexual identity or orientation?)
Sense of self	Addition	Reorder	Reword	Alternative wording concerned distinguishing between self-concept and others’ expectations of self.	“Invalidated self – incongruence between internal self-conceptualisation and the expectations of family, community, culture, religion” (Reword from: How do you feel about yourself?)
	0	0	3		
Loss of hope	Addition	Reorder	Reword	Rewording suggested to be more specific and focused on future.	“What are your hopes and aspirations for the future?” (Reword from: How do you see your future?)
	0	0	1		
Drugs/alcohol	Addition	Reorder	Reword	Suggested additions to the question on drug/alcohol behavior, methods and relational contexts of drug use.	“[ask about].Method of use, IV, smoking, alone, with others, motivation to address their use” (In addition to - Do you use drugs and alcohol?)
	3	0	0		
Family history of mental health condition	Addition	Reorder	Reword	Alternative wording suggestions concerned terminology around diagnosis.	“Has anyone in your family been diagnosed with a mental health condition (as they may not have “suffered”)?” (Reword from: Has anyone in your family suffered from a mental illness?)
	0	0	2		
Family history of suicide/attempt or behavior	0	0	0		
Other known suicide	Addition	Reorder	Reword	Alternative wording suggestions focused on knowledge of someone’s else’s suicide or suicidality.	“.enquiry about whether they may know someone with suicidal history…. [and/or who has died by suicide]” [Reword from: Do you know someone who died by suicide (either personally or someone unknown or another who has had an impact on you?)]
	0	0	1		
Grief and loss	Addition	Reorder	Reword	Alternative rewording for referring to types of loss (including loss of self).	“Grief and loss is also about the loss of self, particularly for someone with chronic pain conditions.” (Reword from: Has someone you cared for, loved, or had a close relationship with died recently or has an anniversary at or around this time?)
	0	0	1		
All/admin	Addition	Reorder	Reword	Reordering suggestion focused on moving some items (from Part B) to Part A; earlier in questioning.	“Some part B items should be placed earlier, especially things like recent changes in irritability, changes in AOD, and sense of connection OR burden on others – (or to at least have several of these added to the summary sheet at the end).”
	0	1	0		

Table 6 shows that participants responded to all seven domains of enquiry of Part C, with most responses received for the first domain, ‘social support/sense of belonging’ (*n* = 12), followed by ‘self-esteem’ (*n* = 10). Requests for ‘additions’ predominated in the ‘social support/sense of belonging’ domain (*n* = 7) and were also observed in the ‘self-esteem’ domain (*n* = 5) with suggestions for ‘rewording’ (*n* = 5). ‘Rewording’ suggestions were the most frequently observed responses across all other domains of enquiry (*n* = 2–3). Only one domain (Religion), included suggestions for ‘reordering’ of items (*n* = 2).

**Table 6:** STARS-p PART C – summary of suggestions for improvement of item wording, language, and ordering.

Domain of Enquiry	Type of change			Summary of suggestions	Example suggestion (quote)
Social Support/ Sense of Belonging	Addition	Reorder	Reword	Alternative wording suggestions mainly for ‘additions’ concerning presence, types and use of social supports. Rewording suggestions included a focus on trusted sources of support	“Are there any issues (political, religious, personal opinions, moral etc) that would stop you from communicating with normally supportive family or friends about your suicidality?” (Addition).
	7	0	5		“Do you have anyone you trust enough to talk or confide in?” (Reword for: Do you feel closely/intimately connected to others?)
Self-Esteem	Addition	Reorder	Reword	Suggestions for additions as well as rewording of items to gain the view of others on personal qualities.	“What would someone say they like about you / you are good at?” (Addition)
	5	0	5		“… what are 3 positive qualities other people would say about you?” (Reword for: Can you list 3 positive qualities about yourself?)
Coping/Problem Solving Ability	Addition	Reorder	Reword	Rewording suggestions focused on eliciting examples about coping.	“Can you think back to a time when you last felt like this? what helped you cope and get through?” (Reword for: How do you usually cope when you ‘do not feel yourself’/feel down and distressed?)
	0	0	2		
Cultural Identity	Addition	Reorder	Reword	Suggestions for additions focused on trauma while rewording focused on understanding experience of culture /services.	“It would be good to elicit whether the individual has experienced trauma, whether they are a refugee” (Addition)
	1	0	3		“Do you have access to culturally appropriate services/ cultural access?” (Reword for: Do you feel that the community supports your cultural identity?)
Religion	Addition	Reorder	Reword	Reordering suggestions focused on addressing both protective and risk dimensions.	“Risk factor as well as protective factor (many religions shame suicide and this can block the conversation about suicide both before and after the fact)” (Reorder)
	0	2	0		
Child Rearing/ Care Taking	Addition	Reorder	Reword	Suggestions for rewording focused on use of more appropriate and sensitive terms.	“The word obligation could be triggering to some and could be part of the risk factors” (Reword for: Do you have meaningful and/or positive obligations to another/others?)
	0	0	2		
Help-Seeking	Addition	Reorder	Reword	Rewording suggestions concerned obtaining evidence of ‘intention’ to seek help.	“How have you sought help from any sources?” (Reword for: Do you feel comfortable seeking help?)
	0	0	2		
Unspecified	Addition	Reorder	Reword	This suggestion is for a newly created item, namely perceived-control.	“Ask about their sense of control in their life, do they feel out of control, do they feel they can regain control?” (Addition)
	1	0	0		

## 4. Discussion

This qualitative enquiry sought the overall perceptions of the STARS-p (14), from those with a lived experience of suicide. A further aim was to elicit lived experience feedback and suggestions for potential improvement in the wording or language used in the STARS-p. We used a deductive approach to qualitative content analysis to address each of these aims. Regarding overall perceptions of STARS-p, participants typically viewed the protocol questions as relevant to the person, who is seen as expert, and that it promotes collaboration, engagement and facilitates transparency. Participants recognized what STARS-p aspires to achieve, in terms of its comprehensive approach, and how it can be more effectively used and administered. Participants also viewed continuity of care and meeting of diverse client needs as fundamental elements and requirements of the STARS-p. Participants suggested improvements for Parts A, B and C of STARS-p; namely, additions, rewording and reordering of content. These important findings will be more specifically addressed in the planned co-design process for implementing these changes to ensure a final improved version of the STARS-p.

### 4.1. Overall view of the STARS protocol

Three main categories depicted participants’ overall perceptions relating to STARS-p, namely STARS philosophy; What STARS aspires to; and Continuity of care and meeting needs. Within the category STARS philosophy, lived experience perceptions of STARS-p were largely reflective of the intended key philosophical underpinnings of STARS-p (32, 33), such as person-centredness; engagement and collaboration; client narrative focus; and facilitation of transparency in the practitioner/client relationship. While it is possible that participants’ perceptions may have been influenced by the information presented to them earlier in the workshop about STARS-p development (including general philosophy), workshop facilitators continually encouraged their open feedback throughout the workshop. As presented, findings revealed constructive (negative) criticism across many questions posed in this study, therefore, we consider the findings to be representative of the participants’ views. Further, these findings reinforce practitioner perspectives on the person-centred approach (15) and are consistent with current best practice recommendations for SRA in the literature (33, 34). These results also highlight how integral these valued qualities are to building a therapeutic relationship conducive to client disclosure around their suicidality (23, 35). More generally, lived experience perspectives support that STARS-p is consistent with its underlying philosophy.

The category What STARS aspires to included sub-categories focused on: importance of the process of administration of the STARS-p; the big picture of a client’s experience; the importance of STARS training for all users; comparisons with other SRA tools; multi-disciplinary/cross service utility; comprehensiveness of the protocol; and availability of the protocol. Notably, these issues identified are well aligned with content of the STARS training (see (32) for outline of modules and topics). The training emphasizes appropriate administration, including adherence to the process, but also a strengths-based approach focusing on client self-determination and client as expert. While STARS-p training was not presented in the workshop to participants, facilitators did convey that STARS-p training was required for administration or use of the protocol. Nevertheless, the present findings reinforce the ongoing emphasis on these elements in the training. Furthermore, these findings align with the STARS-p and training focused on documentation of client informed referral and follow-up responses necessary for multi-disciplinary/cross service utility and meeting of identified psycho-social needs; which is recommended best practice in the literature (36, 37).

The final category Continuity of care and meeting needs revealed key issues reflective of ‘real world’ concerns, with sub-categories depicting delivery time concerns (around completion of the entire STARS interview); setting dependence (applicability across different settings including time-limited consultations); recognizing diversity (e.g., appropriate language use for LGBTIQAP+ and other groups); follow-on to other services (including referral to meet client needs); and using SRA information for planning as to how to meet client needs. These findings highlight concerns about genuinely meeting client needs through the SRA process of the STARS-p, and whether service providers and clinicians will be able to follow these through. While comprehensive psychosocial needs-based assessment can legitimize client distress through exploration of client narrative about their experience (38), less is known about how the outcomes of this process might effectively progress commensurate care responses. The documentation and duty of care module of STARS training (32) is designed to support practitioners to identify effective follow-up care and referral services locally for their clients as part of the SRA process. However, further research is needed to investigate the nature of referrals and care responses implemented subsequent to the STARS-p administration.

### 4.2. Suggestions for refinement and improvements of the STARS-p

Across all parts of STARS-p, the majority of participant suggestions related to ‘rewording’ of items while ‘additional’ items were suggested mostly for Parts B and C, and only a few suggestions concerned ‘reordering’ of items. For example, for Part A (see Table 4), the main suggestions were for rewording of existing terminology, to enhance sensitivity and respectful language, to encourage client disclosure of suicidality. This was particularly so for the item ‘death ideation’ [existing wording – “Have you wished to be dead?”], which one participant suggested being reworded to: “Have you thought about your death or dying in general, such as by natural causes, but not suicide specifically?” Similarly, for Part B of the protocol (see Table 5), most suggestions for rewording of items focused on increasing the sensitivity of questions, such as in the domains ‘sexual identity/orientation’ and ‘sexual/physical/emotional abuse’. For example, “What has your experience of your sexuality and gender been like?” was suggested (in place of “Are you comfortable with your sexual identity or orientation?”). This open question may provide more scope for further exploration and would also support clinicians to adapt aspects of the protocol for diverse populations (‘recognizing diversity’ sub-category). Responses relevant to Part C, likewise, mainly reflected suggestions for rewording of items, along with suggestions for additional items (more so than indicated for Parts A and B). For example, with regards to the self-esteem domain of enquiry, a suggestion was made to add the probe “What would someone [someone you know] say they like about you / you are good at?” Overall, these suggestions for reframing questions focus on empowering the client, reducing judgment, and improving therapeutic alliance. Such values of person-centred practice have previously been reported by those with lived experience, as key to undertaking effective therapeutic psychosocial needs-based assessment (23, 39). However, the current study extends this understanding by providing tangible examples of preferred wording or lines of enquiry regarding suicidality.

### 4.3. Implications

The current findings regarding the suggestions of those with a lived experience of suicide will provide initial information for a deeper re-design process aimed to support the suggested improvements to STARS-p for the subsequent edition. Specifically, the suggestions for rewording, reordering and addition of items will be considered and fine-tuned to enhance the appropriateness of the enquiry domains within each part of STARS-p. This will be achieved through a co-design process involving collaboration between those with a lived experience of suicide and STARS-p researchers (and authors) to refine the wording of questions based on the findings of the present study. Furthermore, it would be useful to gather perspectives from clinicians and coroners regarding certain questions where there is an expected minimum standard duty of care requirement (e.g., the suicide enquiry; Part A) during the redesign process. The reconstruction of questions throughout the protocol is expected to further promote the underlying person-centred philosophy of the new edition of the STARS-p. Similarly, corresponding modifications will be made to the STARS training to improve understanding of, and response to, clients experiencing suicidal distress, by clinicians and other professionals using the STARS-p. Ideally, a review of the updated edition of STARS-p involving those with a lived experience of suicide, is planned, before the STARS-p is rolled-out for dissemination in practice.

### 4.4. Strengths and limitations

The key strength of this study relates to the novel engagement of those with a lived experience of suicide to provide suggestions for informing refinement and improvement of the next edition of STARS-p. It is recognized that it is optimal for co-design processes to occur at the outset of development of SRA approaches. Nevertheless, the present study represents an important step for promoting the collaboration and engagement of individuals with lived experience perspectives in future refinements and adaptations of the protocol. Furthermore, the subsequent re-design phase for implementing the suggestions from this study, will include a wider diversity of lived experiences of suicide, which is an important strength of the planned translation of our findings.

The main study limitations concern the composition and representativeness of the sample and the deductive approach for obtaining qualitative data. Regarding the former, we acknowledge that the demographics of our sample include predominantly middle-aged Caucasian females, which is not representative of the population of those with lived experience of suicide. Our sample was largely influenced by the characteristics of attendees at the National Lived Experience Summit, from which our volunteer participants were recruited. Ideally, inclusion of adults from across the lifespan and of increased gender and cultural diversity would have enhanced the representativeness and broader application of our findings. Additionally, we acknowledge that responses and suggestions made by those with a lived experience of suicide in our study, do not necessarily reflect the perceptions of all individuals with lived experience, since no single experience, and therefore perception, will be the same. Furthermore, the composition of lived experiences in our study may be considered a limitation, since not all participants (approximately 25%) reported having experienced suicidal thoughts/behavior. While most of the sample had experienced suicidal thoughts (79%) or survived an attempt (75%), it may be argued that those who have not, are not able to directly express the lived experience of being asked about their own suicidality. Nevertheless, we purposively also included those who have cared for someone experiencing suicidal distress and/or been bereaved by suicide because we wanted to understand their perceptions concerning appropriate and safe language and questioning based on their support and care for those experiencing suicidal thoughts/behavior. Another limitation concerns our lack of information about whether participants in our sample had been trained in or used STARS-p in practice or had been recipients of STARS-p (as a client).

Furthermore, although the purpose of qualitative research is not to generalize findings to other populations, the process of enquiry used to gather insights from lived experience in this study may be relevant for other researchers and/or authors considering input from lived experience around co-design of existing assessment protocols. Regarding our use of a deductive approach for answering Questions 2–4 of our study, participant feedback around what is important to ask about, and how, may have been limited from the outset by specific and structured questions asked of them, in alignment with pre-existing STARS-p domains of enquiry and probes. Rather, a more open-ended inquiry to understanding lived experience perceptions about exploring suicidality and safety of a person in suicidal distress, may have yielded novel insights regarding important domains of enquiry as well as the format and type of questions to ask concerning these. Nevertheless, given the aim of the study was to improve an existing protocol via a co-design process, rather than develop a new protocol, use of more structured format was deemed the most appropriate for the purpose of this study.

## 5. Conclusion

The current findings provide important directions for the refinement of the STARS-p. Our study yielded invaluable perspectives, from those with a lived experience of suicide, which will inform improvements to the STARS-p as a person-centred, psychosocial needs-based assessment protocol. Further, the importance of appropriate training to support sensitive questioning around suicide has been emphasized as critical to its effectiveness, and as such, STARS training will be updated to align with the important protocol changes expected from this study.

## Data availability statement

The datasets presented in this article are not readily available because the data from this study are available on request from the corresponding author, [JH]. The data are not publicly available due to the sensitivity of some participant responses. The rationale for data requests should not intrude on the privacy of research participants. Requests to access the datasets should be directed to jacinta.hawgood@griffith.edu.au.

## Ethics statement

The studies involving human participants were reviewed and approved by Griffith University Human Research Ethics Committee (Ref number: 2021/651/HREC). The participants provided their written informed consent to participate in this study.

## Author contributions

JH conceptualized the study, with methodological guidance by TO and workshop support by CB and BE, and wrote the original manuscript. JH, TO, KK, CB, and BE analyzed the data. TO, SHS, and KK provided edit and reviews. TO, SHS, EA, and DDL are supervisors of PhD candidate JH. All authors contributed to the final reviewing of the manuscript, and read and approved the submitted version.

## Conflict of interest

The authors declare that the research was conducted in the absence of any commercial or financial relationships that could be construed as a potential conflict of interest.

## Publisher’s note

All claims expressed in this article are solely those of the authors and do not necessarily represent those of their affiliated organizations, or those of the publisher, the editors and the reviewers. Any product that may be evaluated in this article, or claim that may be made by its manufacturer, is not guaranteed or endorsed by the publisher.
