# Supplementary material for: Lived experience perspectives guiding improvements to the Systematic Tailored Assessment for Responding to Suicidality protocol
Source: Front Psychiatry. 2023 Jul 7;14:1074805. doi: 10.3389/fpsyt.2023.1074805 (PMC10361574; doi:10.3389/fpsyt.2023.1074805)

Supplementary Material

# Supplementary Material 1. STARS – Systematic Tailored Assessment for Responding to Suicidality

## Systematic Tailored Assessment for Responding to Suicidality (STARS) protocol was first developed in 2015 by Hawgood and De Leo (Hawgood & De Leo, 2015) as an alternative to actuarial risk tools. It is a person-centered, psycho-social needs-based semi-structured interview (Hawgood & De Leo, 2016). STARS protocol (2^nd^ Edition, 2018) (Hawgood & De Leo, 2018) includes empirically informed questions regarding indicators of suicidality (Part A), risk factors (Part B) and protective factors (Part C), to facilitate exploration of the clients’ experience of suicidality and psycho-social factors. Instead of stratified suicide risk level outcomes however, STARS includes client-rated ‘levels of concern’ associated with elements of the suicidal enquiry, risk factors and protective factors. The client’s narrative about what factors are most concerning to them and therapist judgement are integrated to determine priority foci for determining foreseeability, safety planning and immediate management responses. Finally, within the clinical notes section, the protocol provides for documentation of the client’s suicidal state and psycho-social needs, and importantly, the commensurate actions proposed by the clinician for demonstration of standard of care, colleague consultation and follow-up and documentation of significant other involvement (where applicable) in safety planning (Stanley & Brown, 2012) (see STARS protocol structure below).


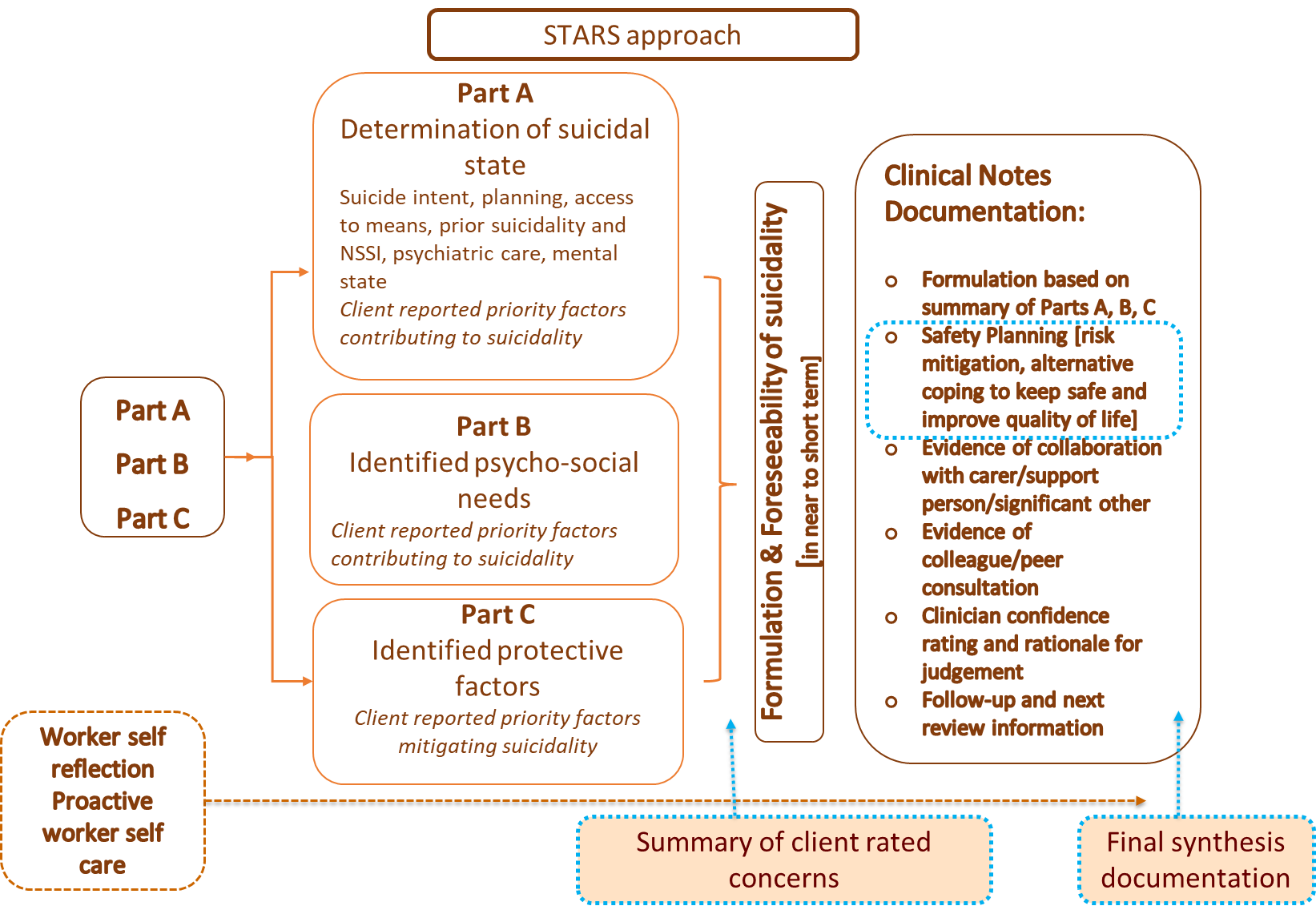

Supplement: Supplementary file 1 [file Table_1.DOCX]
