# Supplementary material for: Lived experience perspectives guiding improvements to the Systematic Tailored Assessment for Responding to Suicidality protocol
Source: Front Psychiatry. 2023 Jul 7;14:1074805. doi: 10.3389/fpsyt.2023.1074805 (PMC10361574; doi:10.3389/fpsyt.2023.1074805)
Supplement: Supplementary file 3 [file Table_3.DOCX]

Supplementary Material

**Supplementary Material 3.** Schedule for STARS Protocol workshop: Enhancing Co-authorship of Commensurate Care

| *Time* | *Topic* | *Content* |
| --- | --- | --- |
| 8.45am |  | Participants enter workshop platform |
| 9am – 9.10am | Introductions (facilitators and background) | Facilitators, acknowledgement of country and lived experience, safety and support/self-care.  Research rationale and critical inclusion of lived experience co-design and participation.  STARS protocol – need for lived experience input |
| 9.10 – 9.55am | STARS protocol: philosophy and approach | STARS background: Philosophy, Medical vs client oriented; psycho-social needs, client narrative and commensurate care outcomes.  STARS Parts A, B, and C: Important domains of enquiry and items of interest |
| 10.00-10.25am | Break out Rooms | - Activity 1: Brainstorm and peer sharing / whiteboard - *Overall view of STARS protocol* |
| 10.25-10.30am | Main room | SLIDO Final answers: Overall view of STARS protocol |
| 10.30 – 11:00am | *Morning Tea Break* | |
| 11.00 – 11.45am | Break out Rooms | - Activity 2: Brainstorm and peer sharing / whiteboard - *Items in STARS Parts A, B, and C* |
| 11.45 – 12.05pm | Main room | SLIDO Final answers: Parts A, B, and C item suggestions |
| 12.05 – 12.25pm | Summary and debrief | Last overall question – SLIDO – Final comments on STARS protocol |
| 12.25 – 12.30pm | Thank you/close | Self-care check-in and thank you |
